# Supplementary material for: A Structural Perspective on the RNA Editing of Plant Respiratory Complexes
Source: Int J Mol Sci. 2022 Jan 8;23(2):684. doi: 10.3390/ijms23020684 (PMC8775464; doi:10.3390/ijms23020684)
Supplement: Supplementary file 1 [file ijms-23-00684-s001.zip › ijms-1538644-supplementary.pdf]

Supplementary File S1.
This file contains the details for all the analyzed RNA edits in complex I, complex III_2 and complex IV. References for each subunit can be found at the end of each subunit table.

Code key:

- M = in the membrane
- A = amino acid properties
- C = restoration to highly conserved residue
- P = proline removal from alpha helix or beta sheet
- K = vicinity of key functional residues (e.g., hydrophilic axis)
- H = in vicinity of heme
- Q = in vicinity of quinone binding site
- KC = in vicinity of K proton channel
- HC = in vicinity of H proton channel
- DC = in vicinity of D proton channel
- Cu = in vicinity of Cu co-factor
- I = at interface with other subunits
- E = potential interaction with other edited residues
- Ph = lack of edit leads to observed phenotype

Species key:

- Pd = *Paracoccus denitrificans*
- RS = *Rhodobacter sphaeroides*
- Sc = *Saccharomyces cerevisiae*
- Yl = *Yarrowia lipolytica*
- Cr = *Chlamydomonas reinhardtii*
- Dm = *Drosophila melanogaster*
- Bt = *Bos taurus*
- Hs = *Homo sapiens*
- At = *Arabidopsis thaliana*
- Vr = *Vigna radiata*
- Zm = *Zea maize*
- Ob = *Oenothera berteroana*
- Bn = *Brassica napus*
- Rs = *Raphanus sativus*
- Ta = *Triticum aestivum*
- Bv = *Beta vulgaris*
- Ac = *Allium cepa*
- Pp = *Physomitrella patens*
- Hv = *Hordeum vulgare*
- Os = *Oryza sativa*
- St = *Solanum tuberosum*
- Ps = *Pisum sativum*
- Gm = *Glycine max*
- Mg = *Magnolia grandiflora*
- Oe = *Olea europaea*

Complex I
NAD1

Position (At)	mt-DNA	Edited to:	Conservation of edited residue outside of plants	In plants	Code
1	T	M	N/A	*At, Zm*	M
56	S	L	*Pd*	*At*	A
89	R	W	*Yl, Dm, Hs*	*At, Vr*	M, A, Ph – GRS1 PPR
103	P	L	*Yl, Hs*	*At*	M, I
126	R	W	*Pd, Yl, Dm, Hs*	*At*	M, A, C, I
164	P	S	*Pd, Yl, Dm, Hs*	*At, Vr*	M, C
165	R	C	N/A	*At, Vr*	M, I
167	S	L	*Pd, Hs*	*At, Vr*	M, A
179	S	F	*Dm*	*At, Vr*	M, A
191	L	F	*Pd, Yl, Hs*	*At*	M
194	R	C	N/A	*At, Vr*	M, K
203	S	F	*Pd, Yl, Dm, Hs*	*At, Zm*	M, A, C, Ph – Dek605 PPR
212	S	L	*Pd, Yl, Dm, Hs*	*At*	A, C, I
225	S	F	*Pd, Yl, Dm, Hs*	*At, Vr*	M, A
242	P	L	*Pd, Dm, Hs*	*At, Vr*	M, P
248	P	L	*Yl, Dm, Hs*	*At, Vr, Zm*	M, Ph – SMK6 PPR
252	P	L	*Pd, Yl*	*At, Vr*	M
275	L	F	*Pd, Yl, Dm*	*At*	M
300	R	W	*Pd, Yl, Dm, Hs*	*At, Vr*	M, A, C, I
310	R	W	N/A	*At, Vr*	M, A, I
313	P	S	N/A	*At*	M, P, I

References

Ding S, Liu XY, Wang HC, et al. *SMK6 mediates the C-to-U editing at multiple sites in maize mitochondria*. J Plant Physiol., 2019. **240**:152992.

Fan K, Peng Y, Ren Z, et al. *Maize Defective Kernel605 Encodes a Canonical DYW-Type PPR Protein that Edits a Conserved Site of nad1 and Is Essential for Seed Nutritional Quality* [published correction appears in Plant Cell Physiol.,2021 Nov 17;**62**(9):1502]. Plant Cell Physiol., 2020. **61**(11):1954-1966.

Maldonado M, Padavannil A, Zhou L, Guo F, Letts JA. *Atomic structure of a mitochondrial complex I intermediate from vascular plants*. Elife, 2020. **9**: e56664.

Xie T, Chen D, Wu J, Huang X, Wang Y, Tang K, Li J, Sun M, Peng X. *Growing Slowly 1 locus encodes a PLS-type PPR protein required for RNA editing and plant development in Arabidopsis*. J Exp Bot., 2016. **67**(19):5687-5698.

NAD2

Position (At)	mt-DNA	Edited to:	Conservation of edited residue outside of plants	In plants	Code
20	S	F	N/A	*At, Ob*	M, A, I, Ph – DEK40 PPR
30	S	F	N/A	*At, Ob*	M, A, I
87	L	F	N/A	*Ob*	M
114	S	F	N/A	*At, Ob*	M, A, E
115	S	F	N/A	*At, Ob*	M, A, E
123	S	F	N/A	*Zm*	M, P
130	P	L	N/A	*At, Zm, Ob*	M, P, I
132	P	S	N/A	*At, Zm, Ob*	M, P
134	R	C	N/A	*At, Zm, Ob*	M, A
141	P	S	N/A	*Zm*	M
143	H	Y	N/A	*At, Zm, Ob*	M, A
145	S	L	N/A	*Ob*	M, A, I
154	P	L	*Pd, Yl*	*At, Ob*	M, P, K, I
177	S	L	*Pd*	*At, Zm, Ob*	M, A, I
186	P	S	*Pd*	*Zm, Ob*	M, P, K
232	S	F	*Pd*	*At, Ob*	M, A
274	S	F	N/A	*At, Zm, Ob*	M, A, K
278	S	L	N/A	*Zm, Ob*	M, A
281	S	F	N/A	*At, Zm, Ob*	M, A
318	P	L	*Pd, Yl, Dm*	*At, Zm, Ob*	M, P
321	H	Y	*Pd, Hs*	*At, Zm, Ob*	M, A
331	R	C	N/A	*At, Zm, Ob*	M, K
332	T	I	N/A	*At, Zm, Ob*	M, A
354	S	L	N/A	*Zm*	M, A, K
364	S	L	N/A	*At, Zm, Ob*	M, A
386	T	I	N/A	*Ob*	M, A, I
387	S	L	*Yl, Hs, Dm*	*At, Zm, Ob*	M, A
427	P	S	N/A	*At, Zm, Ob*	M, P
437	R	C	N/A	*At, Zm*	M, I
444	A	V	N/A	*Zm, Ob*	M, I
477	T	I	*Yl, Hs*	*Zm*	M, A

478	S	L	N/A	*At, Zm, Ob*	M, A
479	S	F	N/A	*At, Zm, Ob*	M, A
481	P	L	N/A	*Zm*	M, I
485	P	S	N/A	*Zm*	M, I
497	S	L	N/A	*At, Zm, Ob*	M, A, Ph – Org1 PPR

References

Binder S, Marchfelder A, Brennicke A, Wissinger B. *RNA editing in trans-splicing intron sequences of nad2 mRNAs in Oenothera mitochondria.* J Biol Chem., 1992. **267**(11):7615-7623.

Giege, P. and A. Brennicke. *RNA editing in Arabidopsis mitochondria effects 441 C to U changes in ORFs.* Proc Natl Acad Sci U S A, 1999. **96**(26):15324-9.

Kim SR, Yang JI, Moon S, Ryu CH, An K, Kim KM, Yim J, An G. *Rice OGR1 encodes a pentatricopeptide repeat-DYW protein and is essential for RNA editing in mitochondria*. Plant J., 2009. **59**(5):738-49.

Morawala-Patell V, Gualberto JM, Lamattina L, Grienenberger JM, Bonnard G. *Cis- and trans-splicing and RNA editing are required for the expression of nad2 in wheat mitochondria.* Mol Gen Genet., 1998. **258**(5):503-511.

Pruchner D, Beckert S, Muhle H, Knoop V. *Divergent intron conservation in the mitochondrial nad2 gene: signatures for the three bryophyte classes (mosses, liverworts, and hornworts) and the lycophytes.* J Mol Evol., 2002. **55**(3):265-271.

Ren RC, Lu X, Zhao YJ, et al. *Pentatricopeptide repeat protein DEK40 is required for mitochondrial function and kernel development in maize.* J Exp Bot., 2019. **70**(21):6163-6179.

Wang Y, Liu XY, Huang ZQ, et al. *PPR-DYW Protein EMP17 Is Required for Mitochondrial RNA Editing, Complex III Biogenesis, and Seed Development in Maize.* Front Plant Sci., 2021. **12**:693272.

NAD3

Position (At)	mt-DNA residue	Edited to:	Conservation of edited residue outside of plants	In plants	Code
3	S	L	N/A	*At, Zm, Bn, Rs, Ta*	M, A, I
9	S	F	*Dm*	*At, Bn, Rs*	M, A, I,
16	P	L	*Dm, Hs*	*Zm, Ta*	M, P
22	P	L	*Pd, Yl*	*Zm, Ta*	M, P, Ph – MEF47/Dek10 PPR
28	P	L	N/A	*At, Zm, Bn, Rs, Ta*	M, Ph
47*	L	F	*Pd, Dm, Hs*	*Zm, Ta*	Q
50*	S	F	*Pd, Yl*	*At, Zm, Bn, Rs, Ta*	A, Q
63	P	L	*Pd, Yl, Dm, Hs*	*Zm*	M, C, P, I
65	P	S	*Pd*	*Zm, Ta*	M, P, I
71	P	F	*Pd, Yl, Dm, Hs*	*At, Vr, Zm, Bn, Rs, Ta*	M, C, P, K, I, E
73	P	L	*Pd, Yl, Hs*	*Zm, Ta*	M, P, K, I
78	S	F	N/A	*Zm, Ta*	M, A, I
84	P	S	*Pd, Yl*	*At, Vr, Zm, Bn, Rs, Ta*	M, I
85	P	L	*Hs*	*At, Vr, Zm, Bn, Rs, Ta*	M, I, Ph – PpPPR_56 PPR
93	S	F	*Pd, Yl*	*Zm, Ta*	M, A, I, E, Ph – EMP21
104	T	F	N/A	*Zm*	M, A , I
107	S	F	*Pd, Yl*	*Ta*	M, A, I, E
116	S	L	*Pd, Yl, Dm, Hs*	*At, Vr, Zm, Bn, Rs, Ta*	M, A, C, I, E
118	R	W	*Pd, Dm, Hs*	*At, Vr, Zm, Bn, Rs, Ta*	M, A, I

* = loop missing in current structure

References

Bentolila S, Elliott LE, Hanson MR. *Genetic architecture of mitochondrial editing in Arabidopsis thaliana.* Genetics, 2008. **178**(3):1693–1708.

Brehme N, Glass F, Jörg A, Takenaka M. *MEF46 and MEF47 are novel specificity factors for RNA editing sites in mitochondrial nad transcripts.* Mitochondrion, 2020. 53:121-127.

Giege, P. and A. Brennicke. *RNA editing in Arabidopsis mitochondria effects 441 C to U changes in ORFs.* Proc Natl Acad Sci U S A, 1999. **96**(26):15324-9.

Gualberto JM, Bonnard G, Lamattina L, Grienenberger JM. *Expression of the wheat mitochondrial nad3-rps12 transcription unit: correlation between editing and mRNA maturation*. Plant Cell, 1991. **3**(10):1109-1120.

Grosskopf D, Mulligan RM. *Developmental- and tissue-specificity of RNA editing in mitochondria of suspension-cultured maize cells and seedlings.* Curr Genet., 1996. **29**(6):556-563.

Handa H. *The complete nucleotide sequence and RNA editing content of the mitochondrial genome of rapeseed (Brassica napus L.): comparative analysis of the mitochondrial genomes of rapeseed and Arabidopsis thaliana.* Nucleic Acids Res., 2003. **31**(20):5907-5916.

Itani K, Handa H*. Rapeseed mitochondrial ccb206, a gene involved in cytochrome c biogenesis, is co-transcribed with the nad3 and rps12 genes: organization, transcription, and RNA editing of the* nad3/rps12/ccb206 locus. Curr Genet., 1998. 34(4):318-325.

Ohtani S, Ichinose M, Tasaki E, Aoki Y, Komura Y, Sugita M. *Targeted gene disruption identifies three PPR-DYW proteins involved in RNA editing for five editing sites of the moss mitochondrial transcripts.* Plant Cell Physiol., 2010. **51**(11):1942-9.

Qi W, Tian Z, Lu L, Chen X, Chen X, Zhang W, Song R. *Editing of Mitochondrial Transcripts nad3 and cox2 by Dek10 Is Essential for Mitochondrial Function and Maize Plant Development.* Genetics, 2017. **205**(4):1489-1501.

Rankin CT, Cutright MT, Makaroff CA. *Characterization of the radish mitochondrial nad3/rps12 locus: analysis of recombination repeats and RNA editing*. Curr Genet., 1996. **29**(6):564-571.

Wang Y, Liu XY, Yang YZ, Huang J, Sun F, Lin J, Gu ZQ, Sayyed A, Xu C, Tan BC. *Empty Pericarp21 encodes a novel PPR-DYW protein that is required for mitochondrial RNA editing at multiple sites, complexes I and V biogenesis, and seed development in maize*. PLoS Genet., 2019. **15**(8):e1008305.

NAD4

Position (At)	mt-DNA residue	Edited to:	Conservation of edited residue outside of plants	In plants	Code
10	S	F	N/A	*At, Bv*	M, A
15	P	L	N/A	*Ta*	M, P
25	T	I	N/A	*At, Ta, Bv*	M, A
26	P	L	*Dm*	*Ta*	M, E
36	P	L	N/A	*At, Ta, Bv*	M, P, I
52	P	S	*Pd, Dm, Hs*	*Ta*	M, P
53	P	L	*Pd*	*At, Ta, Bv*	M, P, I
55	P	L	*Yl*	*At, Ta*	M, E
56	R	W	N/A	*At, Ta, Bv*	M, A, I, E
66	S	F	*Pd, Yl*	*At, Ta, Bv*	M, A
106	S	L	*Pd, Yl, Dm*	*At, Ta*	M, A, I
121	T	I	*Pd*	*At, Ta, Bv*	M, A, E
123	S	F	*Pd*	*Bv*	M, A
126	R	C	N/A	*At, Ta, Bv*	M
134	S	F	*Pd, Yl, Dm, Hs*	*Ta*	M, A, C, K, Ph – Ogr1 PPR
135	R	C	N/A	*At*	M
139	P	L	*Pd, Dm, Hs*	*Ta*	M, Ph – Ogr1 PPR
145	L	F	*Pd, Dm, Hs*	*Ta, Bv*	M, Ph – Ogr1 PPR
146	S	F	*Pd, Yl, Dm, Hs*	*Ta, Bv*	M, C, K
150	P	L	*Pd, Yl, Dm, Hs*	*At, Bv*	M, C, K, I
203	S	L	*Pd, Hs*	*At, Ta, Bv*	M, A
220	S	F	*Pd*	*At*	M, A
256	P	L	*Pd, Hs*	*At*	M, K
262	H	Y	*Pd, Yl, Dm, Hs*	*At*	M, A, C, E
279	S	F	N/A	*At, Bv*	M, A
299	S	L	*Pd, Yl, Dm, Hs*	*At, Bv*	M, A, C, I
326	P	L	*Yl, Dm, Hs*	*Ta*	M
337	P	L	*Pd, Yl, Dm, Hs*	*At, Bv*	M, C, P, K
345	P	S	N/A	*At*	M, P, K
377	L	F	*Pd, Hs*	*At*	M
391	S	L	N/A	*At*	M, A, I

452	S	L	N/A	*At, Bv*	M, A, I, E
458	S	F	*Yl*	*At, Ta*	M, A, I
469	R	W	N/A	*At, Ta*	M, A, I, E
473	H	Y	*Pd*	*At, Ta, Bv*	M, A, I
478	P	L	N/A	*At, Ta, Bv*	M, P, I
480	R	C	N/A	*Bv*	M, I, E

References

Giege, P. and A. Brennicke. *RNA editing in Arabidopsis mitochondria effects 441 C to U changes in ORFs.* Proc Natl Acad Sci U S A, 1999. **96**(26):15324-9.

Itchoda N, Nishizawa S, Nagano H, Kubo T, Mikami T. *The sugar beet mitochondrial nad4 gene: an intron loss and its phylogenetic implication in the Caryophyllales.* Theor Appl Genet., 2002. **104**(2-3):209-213.

Kim SR, Yang JI, Moon S, et al*. Rice OGR1 encodes a pentatricopeptide repeat-DYW protein and is essential for RNA editing in mitochondria*. Plant J., 2009. **59**(5):738-749.

Lamattina L, Grienenberger JM. *RNA editing of the transcript coding for subunit 4 of NADH dehydrogenase in wheat mitochondria: uneven distribution of the editing sites among the four exons.* Nucleic Acids Res., 1991. **19**(12):3275-3282.

Lamattina L, Weil JH, Grienenberger JM. *RNA editing at a splicing site of NADH dehydrogenase subunit IV gene transcript in wheat mitochondria*. FEBS Lett., 1989. **258**(1):79-83.

NAD4L

Position (At)	mt-DNA residue	Edited to:	Conservation of edited residue outside of plants	In plants	Code
1	T	M	N/A	*Bv, Ac*	M, A, I
14	S	F	*Pd, Dm, Hs*	*At, Vr*	M, A, I
16	S	L	*Yl, Dm*	*Bv*	M, A, I
19	R	W	N/A	*At, Bv*	M, A, Ph – Grs1
29	P	L	*Dm*	*At, Vr*	M, I
32	S	L	*Pd, Dm, Hs*	*At, Vr, Bv*	M, A, K, I
34	P	S	*Pd, Dm*	*At, Vr*	M, P, I, K
37	S	L	*Pd*	*At, Vr, Bv*	M, A, P, K, I, E, Ph – SMK6 PPR
44	S	L	*Yl*	*Bv*	M, A, I
53	S	L	*Pd*	*At, Vr, Bv*	M, A, I
60	S	L	*Yl*	*Bv*	M, A, I
63	S	L	N/A	*At, Vr*	M, A, I
66	P	L	*Pd, Dm, Hs*	*At, Vr, Bv*	M, P, I
94	S	F	N/A	*Bv*	M, A

References

Bentolila S, Elliott LE, Hanson MR. *Genetic architecture of mitochondrial editing in Arabidopsis thaliana.* Genetics, 2008. **178**(3):1693–1708.

Ding S, Liu XY, Wang HC, Wang Y, Tang JJ, Yang YZ, Tan BC. *SMK6 mediates the C-to-U editing at multiple sites in maize mitochondria*. J Plant Physiol., 2019. **240**:152992.

Giege, P. and A. Brennicke. *RNA editing in Arabidopsis mitochondria effects 441 C to U changes in ORFs.* Proc Natl Acad Sci U S A, 1999. **96**(26):15324-9.

Kubo, T. & Yamamoto, M. & Mikami, T. *The nad4L-orf25 gene cluster is conserved and expressed in sugar beet mitochondria.* Theoretical and Applied Genetics, 2000. **100:**214-220.

Maldonado M, Padavannil A, Zhou L, Guo F, Letts JA. *Atomic structure of a mitochondrial complex I intermediate from vascular plants*. Elife, 2020. **9**: e56664.

Tsujimura M, Kaneko T, Sakamoto T, et al. *Multichromosomal structure of the onion mitochondrial genome and a transcript analysis*. Mitochondrion, 2019. **46**:179-186.

Xie T, Chen D, Wu J, et al. *Growing Slowly 1 locus encodes a PLS-type PPR protein required for RNA editing and plant development in Arabidopsis.* J Exp Bot., 2016. **67**(19):5687-5698.

NAD5

Position (At)	mt-DNA residue	Edited to:	Conservation of edited residue outside of plants	In plants	Code
52	P	L	*Dm*	*At, Ob*	M, I
81	P	L	*Pd, Yl*	*At, Ob*	M
91	S	F	N/A	*Ob*	M, A
120	L	F	*Pd, Yl, Dm, Hs*	*At, Ob*	M, C
133	S	F	*Hs*	*At, Ob*	M, A
165	T	I	*Hs*	*At, Ob*	M, A, I
169	P	L	*Yl, Dm, Hs*	*Ob*	M, P, I
180	P	L	*Pd, Yl, Dm, Hs*	*Ob*	M, C, P, I
183	S	L	N/A	*At, Ob*	M, A, I
185	R	C	N/A	*At, Ob*	M
200	R	C	N/A	*At, Ob, Pp*	I, Ph – PpPPR_79 PPR
203	F	V	N/A	*At*	I
210	F	R	N/A	*At*	M, A
238	S	L	*Pd, Yl, Hs*	*At, Ob*	M, A
241	R	W	*Pd, Yl, Dm, Hs*	*Pp*	M, A, C, Ph – PpPPR_91 PPR
242	S	L	*Pd, Yl, Dm, Hs*	*At, Ob*	M, A, C, K
255	R	L	*Pd, Yl, Dm, Hs*	*At*	M, A, C
279	P	S	*Dm, Hs*	*At, Ob*	M
288	S	F	N/A	*Ob*	M, A
292	T	M	N/A	*At, Ob*	M, A
467	S	L	*Pd*	*Ob*	M, A
497	P	L	N/A	*At, Ob, Ta, Hv*	M
517	I	T	N/A	*At, Zm, Ta*	M, A, Ph – ppr2263 PPR
523	D	L	N/A	*Ob*	M, A,
527	S	L	*Pd, Yl, Hs*	*At, Ob, Pp, Ta, Os*	M, A, Ph – mpr25 PPR
530	I	F	N/A	*Ob, Ta*	M
551	F	S	*Dm*	*Hv*	M, A
620	S	F	N/A	*Ta, Hv*	M, A
632	S	L	*Hs*	*At, Ta, Hv*	M, A, I
634	P	L	N/A	*Hv*	M, P, Ph

639	S	F	N/A	*At, Ob, Ta, Hv*	M, A, I, Ph – DEK40 PPR
640	R	C	N/A	*At, Ob, Ta, Hv*	M, A, Ph
653	S	L	N/A	*At, Ta*	Ph – MEF46 PPR

References

Brehme N, Glass F, Jörg A, Takenaka M. *MEF46 and MEF47 are novel specificity factors for RNA editing sites in mitochondrial nad transcripts*. Mitochondrion, 2020. **53**:121-127.

Giege, P. and A. Brennicke. *RNA editing in Arabidopsis mitochondria effects 441 C to U changes in ORFs.* Proc Natl Acad Sci U S A, 1999. **96**(26):15324-9.

Knoop V, Schuster W, Wissinger B, Brennicke A. *Trans splicing integrates an exon of 22 nucleotides into the nad5 mRNA in higher plant mitochondria*. EMBO J., 1991. **10**(11):3483-3493.

Maldonado M, Padavannil A, Zhou L, Guo F, Letts JA. *Atomic structure of a mitochondrial complex I intermediate from vascular plants*. Elife, 2020. **9**: e56664.

Ohtani S, Ichinose M, Tasaki E, Aoki Y, Komura Y, Sugita M. *Targeted gene disruption identifies three PPR-DYW proteins involved in RNA editing for five editing sites of the moss mitochondrial transcripts.* Plant Cell Physiol., 2010. **51**(11):1942-1949.

Pereira de Souza A, Jubier MF, Delcher E, Lancelin D, Lejeune B. *A trans-splicing model for the expression of the tripartite nad5 gene in wheat and maize mitochondria.* Plant Cell, 1991. **3**(12):1363-1378.

Ramadan AM, Alnufaei AA, Khan TK, Ali HM, Eissa HF, Hassan SM. *The first report of RNA U to C or G editing in the mitochondrial NADH dehydrogenase subunit 5 (Nad5) transcript of wild barley.* Mol Biol Rep., 2021. **48**(8):6057-6064.

Ren RC, Lu X, Zhao YJ, Wei YM, Wang LL, Zhang L, Zhang WT, Zhang C, Zhang XS, Zhao XY. *Pentatricopeptide repeat protein DEK40 is required for mitochondrial function and kernel development in maize.* J Exp Bot., 2019. **70**(21):6163-6179.

Sosso D, Mbelo S, Vernoud V, et al. *PPR2263, a DYW-Subgroup Pentatricopeptide repeat protein, is required for mitochondrial nad5 and cob transcript editing, mitochondrion biogenesis, and maize growth.* Plant Cell, 2012. **24**(2):676-691.

Toda T, Fujii S, Noguchi K, Kazama T, Toriyama K. *Rice MPR25 encodes a pentatricopeptide repeat protein and is essential for RNA editing of nad5 transcripts in mitochondria*. Plant J., 2012. **72**(3):450-460.

Uchida M, Ohtani S, Ichinose M, Sugita C, Sugita M. *The PPR-DYW proteins are required for RNA editing of rps14, cox1 and nad5 transcripts in Physcomitrella patens mitochondria*. FEBS Lett., 2011. **585**(14):2367-71.

NAD6

Position (At)	mt-DNA residue	Edited to:	Conservation of edited residue outside of plants	In plants	Code
9	P	L	*Yl*	*At, Vr, Zm*	M, P, I, E
18	A	V	*Pd*	*At, Vr*	M, I
30	P	F	N/A	*At, Vr, Zm*	M, P, I, Ph – Grs1 PPR
32	P	L	*Pd*	*At, Vr*	M, P, E
35	R	C	*Dm*	*At, Vr*	M, A, E
49	S	F	*Pd*	*Zm*	M, A, I
54	P	L	N/A	*At, Vr, Zm*	M, P, K, I
57	H	Y	*Pd, Yl, Hs*	*At, Vr, Zm*[4]	M, A, K, I
64	S	L	*Pd, Yl, Dm*	*At, Vr, Zm*	M, A, I
149	S	F	*Pd*	*At, Zm*	M, A, I
190	S	F	N/A	*Zm*	M, A

References

Bentolila S, Elliott LE, Hanson MR. *Genetic architecture of mitochondrial editing in Arabidopsis thaliana.* Genetics, 2008. **178**(3):1693–1708.

Giege, P. and A. Brennicke. *RNA editing in Arabidopsis mitochondria effects 441 C to U changes in ORFs.* Proc Natl Acad Sci U S A, 1999. **96**(26):15324-9.

Haouazine-Takvorian N, Takvorian A, Jubier MF, Lejeune B. *Genes encoding subunit 6 of NADH dehydrogenase and subunit 6 of ATP synthase are co-transcribed in maize mitochondria.* Curr Genet., 1997. **31**(1):63-69.

Maldonado M, Padavannil A, Zhou L, Guo F, Letts JA. *Atomic structure of a mitochondrial complex I intermediate from vascular plants*. Elife, 2020. **9**: e56664.

Xie T, Chen D, Wu J, et al. *Growing Slowly 1 locus encodes a PLS-type PPR protein required for RNA editing and plant development in Arabidopsis.* J Exp Bot., 2016. **67**(19):5687-5698.

NAD7

Position (At)	mt-DNA residue	Edited to:	Conservation of edited residue outside of plants	In plants	Code
13	S	L	*Dm, Hs*	*At, St*	A, I
26	S	L	*Pd, Yl, Dm, Hs*	*At, Vr, Zm, Ta, Os*	A, C, K, Ph – EMP21 PPR
28	S	L	*Yl, Dm, Hs*	*St*	A
46	S	L	*Pd, Yl, Dm, Hs*	*At, Vr, Os*	A, C
67	S	F	*Yl, Dm, Hs*	*At, Vr, Os*	A
82	H	Y	*Dm, Hs*	*At, Vr*	A
84	S	L	*Pd, Yl, Dm, Hs*	*At, Vr*	A, C, I
106	R	C	N/A	*At, Vr*	A, K
112	S	L	*Pd, Yl, Dm, Hs*	*At, Vr*	A, C, K
115	S	L	*Pd, Yl*	*At*	A, K
193	S	L	*Pd, Yl, Dm, Hs*	*At, Vr*	A, C
233	S	L	*Pd, Dm, Hs*	*At, Vr*	A, K, I
242	H	Y	*Pd, Yl, Dm, Hs*	*At, Vr*	A, C, K
245	S	L	N/A	*At, Vr*	A
247	L	F	*Pd, Yl, Dm, Hs*	*At, Vr, Zm*[7]	C, K, Ph – SMK6 PPR
257	R	C	*Pd, Yl, Dm, Hs*	*At, Vr*	C, Ph – SMK1 PPR
279	P	L	*Dm, Hs*	*Vr, Zm, Ta, Os*	P, Ph – SMK1 PPR
353	R	C	*Yl, Dm, Hs*	*At, Vr*	A
360	S	F	*Pd, Yl, Dm, Hs*	*At, Vr*	A, C, I
363	S	L	*Pd, Yl, Dm, Hs*	*At, Vr*	A, C
368	S	F	N/A	*At, Vr*	A
375	P	L	*Pd, Yl, Dm, Hs*	*At, Vr*	C, I

References

Bentolila S, Elliott LE, Hanson MR. *Genetic architecture of mitochondrial editing in Arabidopsis thaliana.* Genetics, 2008. **178**(3): p. 1693–1708.

Carrillo C, Bonen L. *RNA editing status of nad7 intron domains in wheat mitochondria.* Nucleic Acids Res.,1997. **25**(2):403-409.

Ding S, Liu XY, Wang HC, et al. *SMK6 mediates the C-to-U editing at multiple sites in maize mitochondria.* J Plant Physiol., 2019. **240**:152992.

Gäbler L, Herz U, Liddell A, et al. *The 42.5 kDa subunit of the NADH: ubiquinone oxidoreductase (complex I) in higher plants is encoded by the mitochondrial nad7 gene*. Mol Gen Genet., 1994. **244**(1):33-40.

Li XJ, Zhang YF, Hou M, et al. *Small kernel 1 encodes a pentatricopeptide repeat protein required for mitochondrial nad7 transcript editing and seed development in maize (Zea mays) and rice (Oryza sativa).* Plant J., 2014. **79**(5):797-809.

Maldonado M, Padavannil A, Zhou L, Guo F, Letts JA. *Atomic structure of a mitochondrial complex I intermediate from vascular plants*. Elife, 2020. **9**: e56664.

Takenaka M. *MEF9, an E-subclass pentatricopeptide repeat protein, is required for an RNA editing event in the nad7 transcript in mitochondria of Arabidopsis.* Plant Physiol., 2010. **152**(2):939-947.

Wang Y, Liu XY, Yang YZ, et al. *Empty Pericarp21 encodes a novel PPR-DYW protein that is required for mitochondrial RNA editing at multiple sites, complexes I and V biogenesis, and seed development in maize*. PLoS Genet., 2019. **15**(8):e1008305.

NAD9

Position (At)	mt-DNA residue	Edited to:	Conservation of edited residue outside of plants	In plants	Code
5	S	F	N/A	*Ta, Os*	A, I
31	S	F	N/A	*At, Vr, Ta, Os, St*	A
38	P	L	*Pd*	*Ta, Os*	E
56	S	L	*Pd, Dm, Hs*	*At, Vr, Ta, Os, St*	A, E
64	H	Y	*Yl*	*At, Ta, Os*	A, I, Ph – EMP5 PPR
75	H	Y	*Pd, Yl, Dm, Hs*	*Ta, Os*	A, C, E
100	P	S	*Pd, Yl, Dm, Hs*	*At, Vr, Ta, Os, St*	C, E
104	P	L	*Yl, Pd*	*Os*	I
110	R	W	*Pd, Yl, Dm, Hs*	*Vr, Ta, Os, St*	A, C, I, Ph – EMP5 PPR
119	S	F	*Pd, Hs*	*Ta, Os*	A, Ph – EMP5 PPR
123	S	F	*Pd, Yl, Dm, Hs*	*Ta, Os, St*	A, C, E
133	S	L	*Pd, Dm, Hs*	*At, Vr, Ta, St*	A, I
147	L	F	*Pd, Yl, Dm, Hs*	*At, Vr, St*	A, C

References

Bentolila S, Elliott LE, Hanson MR. *Genetic architecture of mitochondrial editing in Arabidopsis thaliana.* Genetics, 2008. **178**(3):1693–1708.

Giege, P. and A. Brennicke. *RNA editing in Arabidopsis mitochondria effects 441 C to U changes in ORFs.* Proc Natl Acad Sci U S A, 1999. **96**(26):15324-9.

Grohmann L, Thieck O, Herz U, Schröder W, Brennicke A. *Translation of nad9 mRNAs in mitochondria from Solanum tuberosum is restricted to completely edited transcripts.* Nucleic Acids Res., 1994. **22**(16): 3304-11.

Lamattina L, Gonzalez D, Gualberto J, Grienenberger JM. *Higher plant mitochondria encode a homologue of the nuclear-encoded 30-kDa subunit of bovine mitochondrial complex I*. Eur J Biochem., 1993. **217**(3):831-838.

Liu YJ, Xiu ZH, Meeley R, Tan BC. *Empty pericarp5 encodes a pentatricopeptide repeat protein that is required for mitochondrial RNA editing and seed development in maize*. Plant Cell, 2013. **25**(3):868-883.

Maldonado M, Padavannil A, Zhou L, Guo F, Letts JA. *Atomic structure of a mitochondrial complex I intermediate from vascular plants*. Elife, 2020. **9**: e56664.

Nishiwaki S, Nakazono M, Tsutsumi N, Hirai A. *Structure of a gene subunit 9 of NADH dehydrogenase (nad9) in rice mitochondria and RNA editing of its transcript*. Plant Cell Physiol., 1995. **36**(6):1135-38.

Wang Y, Liu XY, Yang YZ, Huang J, Sun F, Lin J, Gu ZQ, Sayyed A, Xu C, Tan BC. *Empty Pericarp21 encodes a novel PPR-DYW protein that is required for mitochondrial RNA editing at multiple sites, complexes I and V biogenesis, and seed development in maize*. PLoS Genet., 2019. **15**(8):e1008305.

Complex III_2

COB

Position (Vr)	mt-DNA	Edited to:	Conservation of edited residue outside of plants	In plants	Code
18	T	I	N/A	*St*	M, A
60	H	Y	*Sc, Cr, Dm, Hs*	*Ta*	M, C, H
96	L	F	*Sc, Cr, Dm, Hs*	*At, Vr, Ta, Os, St*	M, C
100	H	Y	*Pd, Yl, Cr, Dm, Bt*	*Vr, Ta, Os, St*	M, A
109	H	Y	*Sc, Cr, Dm, Hs*	*At, Vr, Ta, Os, St*	M, A, C
120	R	W	*Sc, Cr, Dm, Hs*	*Vr, Ta, St*	M, A, C, H
140	P	L	*Pd, Yl, Cr, Dm, Bt, Hs*	*Ta*	H
188	P	L	*Pd, Sc, Yl*	*Ta*	M, P, I
190	H	Y	*Pd, Sc, Yl, Cr*	*At, Vr, Ta, Os, St*	M, A, H, I
194	L	F	*Sc, Cr, Dm, Hs*	*Ta*	M, C, H, I
227	S	F	*Pd, Cr, Dm, Bt, Hs*	*Ta, St*	M, A
239	R	W	N/A	*Ta*	M, A
242	S	F	*Sc, Yl, Hs*	*Ta*	M, A
270	P	S	N/A	*Vr, Ta, Os, St*	Q
285	H	Y	*Sc, Cr, Dm, Hs*	*At, Vr, Ta, Os, St*	M, C, Q
303	P	L	N/A	*At, Vr, Ta, Os, St*	M, P, Ph – EMP5 PPR
328	H	Y	N/A	*At, Vr, Ta, Os, St*	M, A
339	R	C	N/A	*At, Vr*	M
361	P	S	N/A	*Vr, Os, St*	M, P
362	P	S	N/A	*St*	M, P
375	P	L	N/A	*Vr, Ta, Os, St*	M, P

References

Giege, P. and A. Brennicke. *RNA editing in Arabidopsis mitochondria effects 441 C to U changes in ORFs.* Proc Natl Acad Sci U S A, 1999. **96**(26):15324-9.

Gualberto JM, Lamattina L, Bonnard G, Weil JH, Grienenberger JM. *RNA editing in wheat mitochondria results in the conservation of protein sequences*. Nature, 1989. **341**(6243):660-662.

Ito Y, Nakazono M, Kadowaki K, Tsutsumi N, Hirai A. *RNA editing of transcripts of the gene for apocytochrome b (cob) in rice mitochondria.* Genes Genet Syst., 1996. **71**(2):85-89.

Liu YJ, Xiu ZH, Meeley R, Tan BC. *Empty pericarp5 encodes a pentatricopeptide repeat protein that is required for mitochondrial RNA editing and seed development in maize*. Plant Cell, 2013. **25**(3):868-883.

Maldonado M, Guo F, Letts JA. *Atomic structures of respiratory complex III_2, complex IV, and supercomplex III_2-IV from vascular plants.* Elife, 2021. **9**: e56664.

Zanlungo S, Bégu D, Quiñones V, Araya A, Jordana X. *RNA editing of apocytochrome b (cob) transcripts in mitochondria from two genera of plants.* Curr Genet., 1993. **24**(4):344-348.

Complex IV

COX1

Position (Vr)	mt-DNA	Edited to:	Conservation of edited residue outside of plants	In plants	Code
81	S	F	*Pd, Sc, Dm, Bt, Hs*	*At, Zm*	M, A, C, H
85	S	F	*Pd*	*At, Zm*	M, A, H
148	S	L	*Pd, Sc, Dm, Bt, Hs*	*At, Zm*	M, A, C, I
151	S	F	*Pd, Sc, Dm, Bt, Hs*	*At, Zm*	M, A, I
172	S	F	N/A	*At, Zm*	M
192	P	L	*Hs*	*At, Zm*	M
197	P	L	*Pd, Sc, Bt, Hs*	*At*	M, C, P
223	S	F	*Pd, Sc, Dm, Bt, Hs*	vascular and non-vascular plants	M, A, C
239	R	W	*Pd, Sc, Dm, Bt, Hs*	vascular and non-vascular plants	M, A, C, H, KC
249	P	L	*Pd, Sc, Dm, Bt, Hs*	vascular and non-vascular plants	M, H, C, KC
254	S	F	*Pd, Sc, Dm, Bt, Hs*	vascular and non-vascular plants	M, A, C, KC
396	H	Y	*Pd, Sc*	*At, Zm*	M, A, HC
427	L	F	*Pd, Sc, Dm, Bt, Hs*	vascular and non-vascular plants	M, C, H
469	R	C	N/A	*At*	M, HC
497	P	S	*Sc, Hs*	*Zm*	K, I, Ph – SMK4 PPR

References

Jerome Coyne H, Ciofi-Baffoni S, Banci L, Bertini I, Zhang L, George GN, Winge DR. *The Characterization and Role of Zinc Binding in Yeast Cox4**. JBC, 2007. **282**(12):8926-34.

Sper-Whitis, G.L., J.L. Moody, and J.C. Vaughn. *Universality of mitochondrial RNA editing in cytochrome-c oxidase subunit I (coxI) among the land plants*. Biochim Biophys Acta, 1996. **1307**(3):301-8.

Suzuki H, Yu J, Ness SA, O'Connell MA, Zhang J. *RNA editing events in mitochondrial genes by ultra-deep sequencing methods: a comparison of cytoplasmic male sterile, fertile, and restored genotypes in cotton.* Mol Genet Genomics, 2013. **288**(9):445-57.

Wang Y, Liu XY, Huang ZQ, et al. *PPR-DYW Protein EMP17 Is Required for Mitochondrial RNA Editing, Complex III Biogenesis, and Seed Development in Maize.* Front Plant Sci., 2021. **12**:693272.

COX2

Position (At/Vr)	mt-DNA residue	Edited to:	Conservation of edited residue outside of plants	In plants	Code
3	S	L	N/A	*Zm*	N/A
9	L	F	N/A	*At, Zm*	N/A
24	S	F	*Pd, Yl*	*At, Zm*	M, A, I
54	S	L	*Hs*	*Zm, Os*	M, A, Ph – Ogr1 PPR
55	R	W	N/A	*Zm*	M, A, I
85	R	W	*Pd, Yl, Dm, Hs*	*At, Zm, Ob, Ta, Os, Ps, Gm*	M, A, C, I
93	S	L	*Pd, Yl, Dm, Hs*	*At, Zm, Ob, Os, Ps, Gm*	M, A, C, I
123	R	W	*Pd, Yl, Dm, Hs*	*Pp*	A, C, K
125	R	W	*Pd, Yl, Dm, Hs*	*Zm*	A, C, K, Ph – PpPPR_77 PPR
127	R	W	*Pd, Yl, Dm, Hs*	*At, Zm, Ob, Ta, Os, Ps, Gm*	A, C, K, E
148	T	M	*Pd, Dm, Hs*	*Zm*	A, K, Ph – EMP18 PPR
159	S	L	*Yl, Hs*	*At*	A, I
182	P	S	*Yl, Hs*	*Zm*	P, K, Ph – DEK10 PPR
186	P	L	*Hs*	*At, Zm, Ta*	I, K, E
194	S	L	*Yl, Dm, Hs*	*At, Zm, Ob, Ta, Os*	A
205	S	L	*Yl, Dm, Hs*	*Zm, Ta, Os*	A, I
211	S	L	*Yl*	*Ta*	A
226	R	C	*Pd, Yl, Dm, Hs*	*Ta*	C, K, Cu, I, E, Cu
233	T	M	*Pd, Yl, Dm, Hs*	*At, Zm, Ta, Os, Gm*	C, K, Cu, E
241	P	S	*Pd, Yl*	*At*	P
248	R	W	*Pd, Yl, Dm*	*At*	A

References

Covello PS, Gray MW. *RNA editing in plant mitochondria* [published correction appears in Nature 1989 Nov 23;**342**(6248):460]. Nature, 1989. **341**(6243):662-666.

Giege, P. and A. Brennicke. *RNA editing in Arabidopsis mitochondria effects 441 C to U changes in ORFs.* Proc Natl Acad Sci U S A, 1999. **96**(26):15324-9.

Gualberto JM, Lamattina L, Bonnard G, Weil JH, Grienenberger JM. *RNA editing in wheat mitochondria results in the conservation of protein sequences.* Nature, 1989. **341**(6243):660-662.

Kim SR, Yang JI, Moon S, et al. *Rice OGR1 encodes a pentatricopeptide repeat-DYW protein and is essential for RNA editing in mitochondria.* Plant J., 2009. **59**(5):738-749.

Li XL, Huang WL, Yang HH, et al. *EMP18 functions in mitochondrial atp6 and cox2 transcript editing and is essential to seed development in maize.* New Phytol., 2019. **221**(2):896-907.

Ohtani S, Ichinose M, Tasaki E, Aoki Y, Komura Y, Sugita M. *Targeted gene disruption identifies three PPR-DYW proteins involved in RNA editing for five editing sites of the moss mitochondrial transcripts.* Plant Cell Physiol., 2010. **51**(11):1942-1949.

Qi W, Tian Z, Lu L, Chen X, Chen X, Zhang W, Song R. *Editing of Mitochondrial Transcripts nad3 and cox2 by Dek10 Is Essential for Mitochondrial Function and Maize Plant Development.* Genetics, 2017. **205**(4):1489-1501.

Yang AJ, Mulligan RM. *RNA editing intermediates of cox2 transcripts in maize mitochondria*. Mol Cell Biol., 1991. **11**(8):4278-4281.

COX3

Position	mt-DNA	Edited to:	Conservation of edited residue outside of plants	In plants	Code
82	P	L	*Pd, Dm, RS, Hs*	*At, Ta, Mg, Oe*	M, C, P, DC, I, Ph – EMP5 PPR
86	S	F	*Pd, Sc, RS*	*At, Ta, Mg, Oe*	M, A, I, Ph – EMP5 PPR
88	P	L	*Pd, Dm, RS, Hs*	*Mg*	M, C, P, I
102	R	W	*Pd, Sc, Dm, Rs, Hs*	*Mg, Oe*	M, A, C, KC, I
104	S	F	*Pd, Dm, RS, Hs*	*At, Ta, Mg, Oe*	M, A, C, I, Ph – DEK45 PPR
105	S	F	*Sc, Dm, RS*	*At, Ta, Mg, Oe*	M, A, I
138	P	L	*Pd, RS*	*At, Ta, Mg, Oe*	M, P
141	P	L	*Pd, Dm, RS, Hs*	*At, Zm, Pp*	M, C, P, Ph – Mef11 PPR
171	S	L	*Dm, Hs*	*Ta, Mg*	M, P
189	S	F	*Pd, Dm, RS, Hs*	*At, Os*	M, A, C, Ph – PpPPR_77 PPR
245	R	W	*Dm, Rs, Hs*	*At, Vr, Zm, Pp*	M, A, C, Ph – Ogr1 PPR
252	R	W	*Pd, Sc, Dm, RS, Hs*	*At, Ta, Mg, Oe*	M, A, C

References

Kim SR, Yang JI, Moon S, et al. *Rice OGR1 encodes a pentatricopeptide repeat-DYW protein and is essential for RNA editing in mitochondria.* Plant J., 2009. **59**(5):738-749.

Liu YJ, Xiu ZH, Meeley R, Tan BC. *Empty pericarp5 encodes a pentatricopeptide repeat protein that is required for mitochondrial RNA editing and seed development in maize*. Plant Cell, 2013. **25**(3):868-883.

Ohtani S, Ichinose M, Tasaki E, Aoki Y, Komura Y, Sugita M. *Targeted gene disruption identifies three PPR-DYW proteins involved in RNA editing for five editing sites of the moss mitochondrial transcripts.* Plant Cell Physiol., 2010. **51**(11):1942-9.

Ren RC, Lu X, Zhao YJ, et al. *Pentatricopeptide repeat protein DEK40 is required for mitochondrial function and kernel development in maize*. J Exp Bot., 2019. **70**(21):6163-6179.

Verbitskiy D, Zehrmann A, van der Merwe JA, Brennicke A, Takenaka M. *The PPR protein encoded by the LOVASTATIN INSENSITIVE 1 gene is involved in RNA editing at three sites in mitochondria of Arabidopsis thaliana*. Plant J., 2010. **61**(3):446-55.
